# What Has Immunology Brought to Periodontal Disease in Recent Years?

**DOI:** 10.1007/s00005-022-00662-9

**Published:** 2022-10-16

**Authors:** Jan Kowalski, Maciej Nowak, Bartłomiej Górski, Renata Górska

**Affiliations:** Department of Periodontology and Oral Diseases, Medical University of Warsaw, Warsaw, Poland

**Keywords:** Periodontal diseases, Immunology, Biomarkers, Immunomodulation, Oxidative stress

## Abstract

Recent decades have shed a new light on the pathomechanism of periodontal inflammation. While classic periodontology concentrates on biofilm control, oral hygiene improvement, professional tooth cleaning and surgical correction of damaged periodontal tissues, new aspects of the destruction mechanisms are being raised. Among them, the greatest attention is paid to the influence of host response on the clinical manifestations of the disease. Numerous studies have proved that the shift from gingivitis to periodontitis is not a simple progress of the disease, but an event occurring only in susceptible individuals. Susceptibility may result from appearance of local factors facilitating biofilm accumulation and/or maturation, or from systemic features, among which over-reaction and prolonged agitation of non-specific component of inflammatory response is crucial. The present paper summarizes the association between periodontology and immunology and updates the knowledge accrued mostly in the recent years. After a brief explanation of advances in understanding of the disease aetiology, the most studied and potentially viable immunological markers of periodontal disease are presented. Possible new therapeutic strategies, exploiting knowledge about the nature of host response—immunomodulation and reduction of chronic oxidative stress—are also presented.

## Introduction

The twenty-first century has brought a shift of paradigm in approach to the aetiology of periodontal inflammation and its treatment. Since 1960s the association between bacteria and the gingivitis—the superficial form of the disease, affecting gums only—has been proven and established. It was shown that deterioration or lack of oral hygiene leads to enhanced colonization of the gingival sulcus (Löe et al.^1965^). Following maturation and diversification of bacteria leads—together with the mineralization of extracellular matrix, clinically manifesting as calculus—to chronic gingival inflammation (Trombelli and Farina 2013). It was not clear, however, how gingivitis transits into periodontitis. A simple progress of the disease was denied by classical studies in the 1970s and 1980s, conducted on the population of Sri Lankan tea laborers, individuals deprived not only of dental service, but also oral hygiene. Although all of them manifested gingivitis, there was a significant percentage of subjects not suffering from periodontitis. In their case gingivitis became a stable inflammatory condition and stopped its progress (Löe et al.^1986^). It became clear that bacteria are necessary, but not sufficient, to initiate periodontitis. That led to description of the so-called “susceptible host”—an individual, that due to some features of his phenotype is prone to develop periodontal inflammation (Hajishengallis 2014). Several local and systemic factors have been identified that increase the risk of initiation or more severe and rapid progress of periodontitis (Van Dyke and Sheilesh 2005). Almost all of them are connected—directly or indirectly—with immune response. The present etiological model of the disease states that periodontal conditions may be stable or unstable. Health is characterised by equilibrium between bacterial load and immune response. An imbalance on the scales leads to progress of periodontal disease. Since such disequilibrium may be caused by either an increase of quality/quantity of microbiota, or a disruption of host defence mechanisms, the new concept of periodontitis involves an immunoinfective etiological model (Loos and van Dyke 2020). It acknowledges what was already obvious from observations—it is unlikely that infection of a pocket would directly lead to destruction of periodontal tissues, because microbiota are natural cohabitants of the oral cavity. In the oral cavity there are 50–100 billion bacteria, over 700 bacterial species in the mouth are identified (Krishnan et al. 2017)—sterilization of the mouth is not only impossible, but potentially harmful. Therefore, attention has been paid to the other weighing pan. Numerous reports have shown that the non-specific component of the immune response, induced by bacteria, is responsible for destruction of periodontal tissues (Van Dyke 2008). Moreover, it has been clearly shown that this exact mechanism is much more important—in terms of pathogenicity and harm for periodontal tissues—than direct toxicity of bacterial enzymes or metabolites (Cekici et al. 2014; Hajishengallis 2014; Van Dyke 2008). Bacteria, apart from secreting numerous enzymes, metabolites and proteins disturbing homeostasis, induce response from neutrophils and macrophages. Since non-specific component relies on the release of reactive oxygen species and degrading enzymes, damage is done both to the microbiota and periodontal tissues. Moreover, lipopolysaccharide fragments of the destroyed bacteria are the strong inducers of the inflammatory response, which further aggravate the damage. Hence, a kind of over-reactive response occurs, which is more significant due to the chronic character of the process and constant supply of the bacteria (Jiao et al. 2014). Referring to the previously mentioned balance status in health and susceptible host concept, some bacteria—regarded as eubiotic microbiota—cause such reaction in a small amount, that results in a clinically unnoticeable immune reaction. Others, however—specifically in the presence of local predisposing factors or systemic features of the host organism—can form a dysbiotic biofilm and may initiate and develop over-reactive and prolonged response of the immune system, that will lead to a periodontal breakdown, clinically manifesting as periodontitis.

## Markers of the Disease

Studies on immune response in periodontal tissues and potential immunomodulative treatment became an important area of periodontal research. The first commercially available genetic test was developed almost 25 years ago. It based on the discovery that individuals bearing mutated allele encoding interleukin (IL)-1 gene cluster produced much more of this cytokine in response to a fixed bacterial load than persons with common allele. In the first published study, non-smoking individuals with rare allele had over 19-fold higher ratio to manifest severe periodontitis than persons with common allele (Kornman et al. 1997). Although further studies did not report such unanimous results, the link between gene polymorphisms, host response level and periodontal inflammation was proven (Huynh-Ba et al. 2007). The test itself was regarded as a predictor of possible future periodontitis (Rudick et al. 2019). There were numerous studies on other possible polymorphisms, such as Fcγ, interferon γ and IL-13, connected with periodontal symptoms (Chai et al. 2012; Shi et al. 2017; Wang et al. 2017; Zhang et al. 2018a). A search for potential markers of periodontitis onset or progression resulted in many potential candidates for such agent—among others matrix metalloproteinase (MMP)-8, IL-1, IL-6, tumor necrosis factor (TNF)-α, PGE_2_ (Barros et al. 2016; Chen et al. 2019; He et al. 2018; Isola 2021). The goal was to be able to quickly and efficiently screen the population to reach individuals requiring more frequent and thorough prophylactic actions, on the other hand reducing clinical efforts put on the rest of the population, not needing so frequent meetings with periodontists or hygienists. The biomarker source would be crevicular fluid of gingival sulcus (gingival crevicular fluid, GCF), scarce serum transudate from the pocket (more abundant during inflammation) (Barros et al. 2016), or saliva, much easier to obtain in relatively large quantities (He et al. 2018). Studies have shown that biomarkers were present in GCF and saliva long before any clinically visible effects of periodontal tissue destruction. So, establishing a viable biomarker of periodontal disease would have a great impact on the disease prevention. An example of such chemical process is increased alkalization of a diseased site. A rise of pH in a pocket to the value of 8.5 is the result of elevated ammonium levels, being a metabolite of protein degradation by periopathogens. Such a rise promotes precipitation of calcium carbonate from saliva or GCF and calculus formation (Barros et al. 2016). Experimental modelling of periodontal disease was able to be processed in an environment able to control multiple risk factors due to an animal model. The protocol of ligation of periodontal tissues in rats has been modernized, described and evaluated (Lin et al. 2021). Further on, results obtained in studies on animals were verified clinically in research, and data were subjected to reviews and meta-analyses. According to the studies, over 90 possible biomarkers have been evaluated in GCF (Loos and Tjoa 2005). Among those are markers directly related to immune reactions. The most extensive research regarded endogenous MMPs, as proteolytic enzymes directly related to periodontal tissue destruction. The influence of MMP-8 on the collagen structure and healing processes was observed on the animal model (Gajendrareddy et al. 2013). Salivary levels of MMP-8 were evaluated in a meta-analysis assessing ten studies on 864 individuals overall. Eight of those studies showed significant elevation of MMP-8 in saliva of periodontal patients, two studies showed opposite results (Zhang et al. 2018b). A South Korean study assessed the influence of non-surgical periodontal treatment on MMP-8 and IL-1β levels in saliva (Kim and Kim 2020). They did not find a statistical difference for either of those two cytokines, although it must be noted that due to heterogeneity only two studies were qualified for the meta-analysis (Kim and Kim 2020). Ghassib et al. (2019) did not include MMP-8 in the meta-analysis presented in their manuscript, although they stated that literature review shows MMP-8 as a valid prognostic marker of periodontal inflammation. A review of 61 articles regarding potential use of MMP-8 as a biomarker of periodontitis shows this enzyme as a strong candidate for an indicator of periodontal inflammation (Al-Majid et al. 2018). Another review, performed by Brazilian researchers, also confirmed the potential of MMP-8 as a prognostic marker for development of periodontitis (De Morais et al. 2018). A cross sectional evaluation of MMP-9 in saliva performed by South Korean researchers proved to be effective in periodontitis screening (Kim et al. 2020). The next group of markers would be inflammatory mediators, such as TNF-α, IL-6, or previously mentioned IL-1. Studies on animals have shown that the antagonists for both IL-1 and TNF significantly reduce recruitment of inflammatory cells in bone proximity (Assuma et al. 1998). Slovenian researchers observed the effect of subcutaneous administration of recombinant human TNF-α on experimental periodontitis in rats, and noticed statistically significant synergistic effect of ligature irritation of periodontium and TNF-α administration, while neither of those treatments alone resulted in a significant increase of periodontal breakdown (Gaspersic et al 2003). A meta-analysis of nine papers regarding patients with periodontitis and type 2 diabetes mellitus showed significantly higher levels of IL-1β in gingival crevicular fluid in those patients compared to the ones with periodontitis alone. No such phenomenon was observed for IL-6 and TNF-α (Atieh et al. 2014). Similar results were reported by Stadler et al. (2016) in their meta-analysis, where elevated levels of IL-6 were also observed. Caldeira et al. (2021) evaluated gene expression of IL-1 and IL-6 in gingival tissues and relevant protein levels in gingival crevicular fluid, and confirmed a rise of both markers in the course of inflammation. A cross-sectional study by Ebersole et al. (2013) conducted on 80 individuals has shown the significant elevation of MMP-8 and decrease of IFN-α in periodontitis patients compared to healthy persons (up to 13-fold and 9-fold, respectively). The mediators mentioned above are related with the connective tissue degradation. A particle associated with bone resorption is prostaglandin E2. It was also evaluated in the cited study, showing a statistically significant, though not so high, increase in periodontitis patients (Ebersole et al. 2013). Another set of mediators related to bone metabolism seems to be even more promising. Ligand of receptor activator of NF-κB (RANKL) is on osteoclasts and its activation is associated with bone resorption. Osteoprotegerin (OPG) is a blocker of this receptor, being produced by—among others—osteoblasts. Studies conducted on animals proved that delivery of OPG inhibits resorption of the alveolar bone in an experimental ligature-induced model (Jin et al. 2007). According to studies on humans, the RANKL/OPG ratio turned out to be a reliable marker of the processes occurring in periodontium (Caldeira et al. 2021). Elevation of RANKL with simultaneous decrease of OPG is observed in periodontitis, whereas the opposite phenomenon occurs in healthy periodontium (Belibasakis and Bostanci 2012; Caldeira et al. 2021) or during the healing process (López Roldán et al. 2020). There are also biomarkers related to oxidative stress, whose role will be explained further. A recent meta-analysis of 32 articles, although suffering from high heterogeneity, showed elevated malondialdehyde both in saliva and GCF when comparing periodontitis with healthy periodontium (Chen et al. 2019). The previously mentioned markers are presented in Table 1.

**Table 1:** Chosen biomarkers for periodontal inflammation, their main function, measured clinical periodontal parameters and the referring articles mentioned in the text

Abbreviation	Full name	Main function		Results	References
MMP-8	Matrix metalloproteinase-8	Degradation of the type I, II and III collagen fibers in the connective tissue		SMD for MMP 1.195 (95% CI 0.72, 1.67)	Meta-analysis Zhang et al. (2018b)
				SMD for MMP 35.90 (95% CI − 31.52, 103.33)	Meta-analysis Kim and Kim (2020)
				N/A (61 studies reviewed)	Systematic review Al-Majid et al. (2018)
				N/A (6 studies reviewed)	Systematic review de Morais et al. (2018)
MMP-9	Matrix metalloproteinase-9	Degradation of the type IV and V collagen fibers in the connective tissue		Salivary levels in ng/mL 283.5 vs 52.6 (periodontitis vs health), *p* < 0.0001	Cross-sectional study Ebersole et al. (2013)
IL-1	Interleukin 1	Stimulation of the non-specific component of the inflammatory response, induced by the Nfkappaβ		Salivary levels in ng/ml 370.7 vs 191.9 (periodontitis vs health), *p* = 0.001, after adjustment for risk factors of periodontitis 16.7 vs 12.5 (respectively), *p* = 0.016	Cross-sectional study Kim et al. (2020)
				Mean difference (diabetics with periodontitis vs nondiabetics with periodontitis) 0.90 (95% CI 0.39, 1.41)	Meta-analysis Atieh et al. (2014)
				IL-1 raised in GCF of periodontal patients (SMD 1.43; 95% CI 0.93, 1.92)	Meta-analysis Stadler et al. (2016)
				IL-1β raised in peri-implant mucositis (SMD 1.94; 95% CI 0.87, 3.35) and peri-implantitis (SMD 2.21; 95% CI 1.32, 3.11)	Meta-analysis Ghassib et al. (2018)
				IL-1β mRNA raised in gingival tissue (mean difference 3.62; 95%CI: 3.43, 3.81), IL-1β raised in GCF (mean difference 95.94; 95% CI 81.96, 109.92)	Meta-analysis Caldeira et al. (2021)
IL-6	Interleukin 6	Promotion of the osteoclasts’ formation		Salivary levels in ng/mL 90.9 vs 7.2 (periodontitis vs health), *p* < 0.0001	Cross-sectional study Ebersole et al. (2013)
				No significant difference for diabetics with periodontitis vs nondiabetics with periodontitis: 0.70 (95% CI − 0.70, 1.41)	Meta-analysis Atieh et al. (2014)
				IL-6 raised in GCF of periodontal patients (SMD 1.64; 95% CI 0.66, 2.63)	Meta-analysis Stadler et al. (2016)
				IL-6 raised in peri-implant mucositis (SMD 1.17; 95% CI 0.16, 3.19) and peri-implantitis (SMD 1.72; 95% CI 0.56, 2.87)	Meta-analysis Ghassib et al. (2018)
				IL-6 mRNA raised in gingival tissue (mean difference 5.42; 95% CI 5.15, 5.68), IL-6 raised in GCF (mean difference 3.63; 95% CI 3.03, 4.23)	Meta-analysis Caldeira et al. (2021)
TNF-α	Tumor necrosis factor alpha	Stimulation of the non-specific component of the inflammatory response, strong chemoattractant		Salivary levels in ng/mL 35.6 vs 3.3 (periodontitis vs health), *p* < 0.0001	Cross-sectional study Ebersole et al. (2013)
				No significant difference for diabetics with periodontitis vs nondiabetics with periodontitis: 0.33 (95% CI − 0.19, 0.86)	Meta-analysis Atieh et al. (2014)
				TNF-α raised in peri-implant mucositis (SMD 3.91; 95% CI 1.13, 6.70) and peri-implantitis (SMD 3.78; 95% CI 1.67, 5.89)	Meta-analysis Ghassib et al. (2018)
PGE_2_	Prostaglandin E2	Stimulation of osteoclast activity		Salivary levels in ng/mL 5.4 vs 1.9 (periodontitis vs health), *p* = 0.07	Cross-sectional study Ebersole et al. (2013)
RANKL	Receptor Activator of Nuclear factor Kappa-B Ligand	Stimulation of osteoclast activity		Salivary levels in ng/mL 226.1 vs 180 (periodontitis vs health), *p* = 0.91	Cross-sectional study Ebersole et al. (2013)
				N/A (11 studies reviewed)	Systematic review Belibasakis and Bostanci (2012)
				Elevation of RANKL in GCF in periodontitis vs health (mean difference 0.32; 95% CI 0.20, 0.43)	Meta-analysis Caldeira et al. (2021)
OPG	Osteoprotegerin	Blocker of RANK		Levels in pg/ml measured at periodontitis and healthy sites 95.5 vs 56.5, respectively, *p* < 0.001. After treatment 68.9 vs 60.6, difference statistically non-significant	Longitudinal study Lopez Roldan et al. (2020)
				N/A (11 studies reviewed)	Systematic review Belibasakis and Bostanci (2012)
MDA	Malondialdehyde	Product of the peroxidation of polyunsaturated fatty acids		Levels in pg/ml measured at periodontitis and healthy sites 3.1 vs 7.0, respectively, *p* < 0.001. After treatment 6.6 vs 6.7, difference statistically non-significant	Longitudinal study Lopez Roldan et al. (2020)
				SMD for salivary MDA 1.74 (95% CI), SMD for MDA in GCF 2.86 (95% CI)	Meta-analysis Chen et al. (2019)

*SMD* standardized mean difference, *CI* confidence interval, *GCF* gingival crevicular fluid

The role of immune response in periodontal disease process was even more recognized with the classifications of periodontal diseases basing directly on antibodies’ levels. In 2007, Prof. Offenbacher conducted a research on 6700 elderly patients and grouped them according to seral immunoglobulins against eight periopathogens. It was found that patients could be grouped due to clusters of the antibodies’ levels, which then correlate with selected clinical periodontal parameters, specifically pocket depth (distance from gingival margin to the base of the pocket formed by gingiva surrounding a given tooth) and bleeding on probing (provoked exudation of blood from the pocket measured by a blunt periodontal probe). The classification, although finally not implemented in general use, was—to the authors’ knowledge—the first attempt to describe periodontal disease severity not solely by parameters measured in the mouth, but also by strength of the immune response (Offenbacher et al. 2007).

## Immunomodulation

Understanding the importance of non-specific inflammatory reaction in the pathogenesis of periodontal inflammation resulted in a shift of therapeutic strategies. The standard approach is aimed at improvement of oral hygiene procedures and professional tooth cleaning (which should result in elimination the biofilm and continuous control of bacterial colonization). Recently, periodontologists have also turned their attention to immunomodulation (Preshaw 2018). As described before, lack of hygiene—and following bacterial colonization of gingival pockets—always results in gingivitis (Löe et al. 1965), and not in all individuals, gingivitis progresses into periodontitis (Löe et al. 1986). It was assumed that in the subpopulation of “susceptible individuals” either local factors facilitate bacterial infection, or systemic factors impair immune response, resulting in “hypersensitivity” of the non-specific inflammatory component. The former may be relatively easily managed by dental personnel. The latter requires medications suppressing over-reaction of neutrophils and macrophages, and neutralization of their activity. The so-called “host modulation therapy” includes usage of a wide variety of anti-inflammatory and immunomodulating medications, which will be described below.

MMP’s take a part in degradation of connective tissues. MMP inhibitors were, therefore, regarded as a convenient therapy for modulation of non-specific inflammatory reaction. Doxycycline turned out to be effective in this field. Despite of its antibacterial properties, it also supresses the activity of neutrophil collagenase. This function is also active in smaller doses of doxycycline (20 vs 100 mg used in antimicrobial therapy). Moreover, doxycycline was much more effective in supressing MMP-8 and MMP-9 (secreted by neutrophils) than MMP-1 (secreted by fibroblasts). It led to the assumption that doxycycline is much more specific in modulating inflammation-derived degradation of collagen than physiological turnover of tissues. This stood behind the introduction of subantimicrobial dose doxycycline (SDD) in periodontal therapy (Preshaw et al. 2004). The 20 mg doxycycline (Periostat) for 20 years has been used in the therapy. A review of three studies (92 patients total) showed SDD to be effective in reduction of major clinical periodontal parameters, though the authors reported a high risk of bias and concluded with a call for further, more thorough evaluation (Sgolastra et al. 2011). The panel report of the American Dental Association published four years later confirmed the efficacy of SDD among other adjunctive therapies of periodontal inflammation (Smiley et al. 2015). The most recent analysis was published in 2020, and evaluated SDD together with other adjunctive periodontal therapies (Donos et al. 2020). SDD was evaluated basing on seven studies, five of which were included into the meta-analysis. The authors concluded that SDD was effective in reducing clinical periodontal parameters. In moderate forms of the disease (stage I and II of periodontitis), the achieved progress, however, still statistically significant, was relatively small. The authors stated that the risk of developing drug resistance and treatment costs do not justify its usage in mild forms of periodontitis (stages I and II) and recommend its introduction as an adjunctive agent in stages III and IV of periodontal inflammation (Donos et al. 2020). In another study Trombelli et al. (2020) compared SDD with photodynamic therapy—local release of reactive oxygen species after treating the applied photosentisizer with specific light frequency. The authors did not find any differences between those two additional methods of non-surgical periodontal treatment (Trombelli et al. 2020). Soon after the European Federation of Periodontology (EFP) published its recommendation for treatment of stages I–III of periodontitis. Its workgroups also evaluated host-modulation agents proposed as supportive periodontal therapeutics. EFP suggested not to use SDD as an adjunct to mechanical instrumentation due to a reported risk of liver enzymes’ elevation, unavailability of SDD in several European countries, and the abovementioned risk of triggering bacterial immunity (Sanz et al. 2020). Nevertheless, EFP acknowledged that SDD is particularly effective in severe conditions (where pocket depths reach 7 mm or more) (Sanz et al. 2020).

Non-steroid anti-inflammatory drugs (NSAIDs) constitute another group of immunomodulating medications, widely used in multiple medicine disciplines not only used as anti-inflammatory substances, but also as analgesics or antipyretics. The rationale standing behind the use of NSAIDs in periodontology was their ability to inhibit prostaglandins, which are known to mediate bone resorption. Yet in the 1980s, cross-sectional studies revealed that patients suffering from musculoskeletal disorders and due to this, taking NSAIDs for the prolonged time had better periodontal status than generally healthy individuals (Waite et al. 1981). NSAIDs were used in treatment of periodontitis with relatively good results for periodontal tissues. However, also multiple adverse effects characteristic for NSAIDs were noted, including gastrointestinal problems, tendency for prolonged bleeding, reduced renal perfusion with subsequent fluid retention, and allergic reactions. The abovementioned panel of EFP experts recommending treatment protocols for stages I–III of periodontitis (Sanz et al. 2020) also evaluated the efficacy of NSAIDs, both used systemically and locally. For both local and systemic usage, two randomized-controlled trials were evaluated (Flemmig et al. 1995; Heasman et al. 1993; Oduncuoglu et al. 2018; Yen et al. 2008). All source studies reported improvement of periodontal parameters (namely pocket depth) after NSAIDs usage, no meta-analysis was performed though, due to heterogeneity. A high risk of bias was also reported, as none of the studies provide information on sample size calculation or were underpowered, all of them also declared industry funding (Sanz et al. 2020). The EFP workgroup recommended not to use NSAIDs in the therapy, due to relatively small benefits reported in the studies with high burden of bias, which together with a high risk of drug side effects does not validate recommending them in periodontal therapy (Sanz et al. 2020).

Non-specific inflammatory response is a multi-levelled and multi-branched process. It seems rational to suspect that the earlier an immune reaction is modified (namely suppressed), the more prominent the effect of modification is. In 2017 metabolites of polyunsaturated omega-3 fat acids (PUFA) were described as substances supressing inflammatory reactions (Serhan 2017). They are secreted since the initiation of immune response and serve as negative feedback of inflammatory reaction. Their main function is shifting neutrophil phenotype from active to passive—reducing chemotaxis, inducing elimination of neutrophils from inflamed tissues, and enhancing tissue regeneration. Since they resolve inflammation, they were called resolvins (Abdolmaleki et al. 2020). Their use in periodontal therapy is still in the phase of preclinical studies, but a published systematic review shows that initial results of experiments on animal models are encouraging (Chiang and Serhan 2020) and let believe that periodontal therapy, as well as many other medical branches, will soon be enriched with a new family of efficient medications. For now, an initial evaluation of the influence of PUFA as adjuncts for periodontal treatment did not bring unanimous conclusions (Sanz et al. 2020). Though each of the studies under evaluation reported additional reduction of pocket depth after adding PUFA to treatment protocols, scarce evidence and heterogeneity did not allow the workgroup to draw any concluding remarks (Sanz et al. 2020). Some hopes were associated with bisphosphonates—inhibitors of osteoclastic activity used in treatment of osteoporosis. There were, however, numerous reports of an increased risk of osteonecrosis connected with bisphosphonate intake. Due to the degree of risk and its frequency bisphosphonates are not recommended to be used as a supportive medication in periodontology (Sanz et al. 2020). In Table 2, relevant numeric data from the mentioned literature are presented.

**Table 2:** Chosen immunomodulators utilized in periodontal therapy, mechanism of action, measured clinical periodontal parameters and the referring articles mentioned in the text

Name	Action	Results	References
Doxycycline	Inhibition of MMP-8 and MMP-9 in subantimicrobial dose (20 mg)	MD for use of doxycycline 0.88 mm for CAL (95% CI), 0.64 mm for PD (95% CI)	Meta-analysis Sgolastra et al. (2011)
		MD for use of doxycycline and PD reduction 0.30 mm for moderate pockets (95% CI) and 0.62 mm for deep pockets (95% CI)	Meta-analysis Donos et al. (2020)
		No difference in CAL, PD or BoP between SDD and PDT	Meta-analysis Trombelli et al. (2020)
NSAIDs	Inhibition of prostaglandins	PD for individuals taking NSAIDs vs controls: 1.68 vs 1.95 mms, *p* < 0.01	Cross-sectional study Waite et al. (1981)
		1% flurbiprofren toothpaste twice daily vs placebo: significant greater proportion of sites with bone gain (8.0 vs 3.3%), no differences in sites with bone loss or no change	Randomized-controlled trial Heasman et al. (1993)
		0.3% acetylsalicylic acid topically: significant reduction of PD (median 0.26 mm) compared to controls. No significant difference for BoP	Randomized -controlled trial Flemmig et al. (1995)
		Celecoxib 200 mg daily: significant reduction of PD and CAL in moderate and deep pockets, compared to controls (3.84 vs 2.06 and 3.74 vs 1.43 mms, respectively)	Randomized -controlled trial Yen et al. (2008)
		Diclofenac 50 mg twice daily: significant reduction of PD and attachment gain compared to controls (2.69 vs 2.11 and 2.35 vs 2.12 mms, respectively). Significant reduction of prostaglandin E2 in the GCF (0.06 in logarithmic scale vs no change in controls)	Randomized-controlled trial Oduncuoglu et al. (2018)
Resolvins	Reducing activation of neutrophils	N/A	Systematic review

*NSAIDs* non-steroid anti-inflammatory drugs, *MD* mean difference, *CI* confidence interval, *CAL* clinical attachment loss, *PD* pocket depth, *BoP* bleeding on probing, *SDD* subantimicrobial-dose doxycycline, *PDT* photodynamic therapy

## Advances in Immunomodulation Therapy in Periodontitis

The described medications and substances do not exhaust the topic of potential modulation of inflammatory response. Each month brings new research on this matter, and each of studied topics has a potential to change periodontal therapy and bring it to a new level. A recent review by Chinese authors concerns the use of exosomes. Those vesicles containing any possible particles or substances play the role of intercellular mediators, and may act as a specific and precise regulator of the level of inflammatory response (Lin et al. 2022). Similar data are presented in the study of Kang et al. (2022), who studied the effect of TNF-α on mesenchymal stem cells, in particular the composition of mRNA in exosomes. The results of the study show that treating stem cells with TNF-α enhances immunomodulatory properties of mRNA contained in extracellular vesicles (Kang et al. 2022). A recent study reveals that a subtype of those cells, conditioned with curcumin, may significantly alter immune response. Curcumin, via pathways of extracellular signal-regulated kinase and mTOR (mammalian target of rapamycin), can upregulate cell activity and block prostaglandin E2 activation (Arora et al. 2022). Refined usage of specific stem cells or bacteria can locally change immunological conditions and help specifically design tissue rebuilding and healing processes in the periodontal compartment (Yang et al. 2021). Potential usage of vectoring viruses and gene modification in immunomodulation is, at this moment, problematic due to distortion of immune response by vectors themselves (Evans 2012).

## Chronic Oxidative Stress Reductors

On the molecular level, non-specific inflammatory response leads to release of reactive oxygen species (ROS). Generally, ROS play the role of protectors against bacterial invasion, as they cause death of microorganisms. In the process of periodontal disease, neutrophils release oxygen radicals in an excessive and prolonged manner. This is partially the result of the constant presence of bacterial biofilm, on the other hand—of its composition as maturating biofilm contains dysbiotic bacteria, which have the ability to strongly induce non-specific component of the inflammatory response (Chapple et al. 2007). A prolonged and sustained release of ROS results in chronic oxidative stress—inability of an organism to neutralise ROS arising from an imbalance between antioxidants and oxygen free radical (Chapple et al. 2007). This leads eventually to degradation of surrounding tissues, clinically manifesting as progressing periodontal disease. Reducing chronic oxidative stress in modern periodontology can be conducted both in therapy, as a support for the classical instrumentation, or in the maintenance phase, with proper dietary habits preventing periodontal disease relapse. The candidate substance is resveratrol—a polyphenol naturally present in the skin of dark grapes, cranberries and red wine. It was shown to promote fibroblast activity and inhibit bone resorption in a rat model (Bhattarai et al. 2016). Studies in vitro have shown the ability of resveratrol to neutralize pathological properties of *Porphyromonas gingivalis*, one of the major periopathogens. Resveratrol inhibited formation of biofilm, reduced adherence of bacteria to protein matrix, attenuated induction of the NF-κB pathway and inhibited *P. gingivalis* protease activity (Ben Lagha et al. 2019). Another potential antioxidant is curcumin—a dietary spice. An animal model has shown curcumin to decrease osteoclastic activity marked by RANKL/OPG ratio (Xiao et al. 2018).

The abovementioned findings were also implemented in so-called “healthy periodontal diet”, low in carbohydrates, but rich in PUFA and antioxidants. A randomized controlled study reported beneficial influence of such nutrients on the clinical periodontal parameters (Woelber et al. 2016). Also, kiwi, fruit very rich in vitamin C, was reported in another randomized trial as able to reduce gingival inflammation index when eaten in the amount of two pieces per day for five months (Graziani et al. 2018). Those results suggest that low-sugar food, rich in omega-3 acids and antioxidants would prove beneficial not only for periodontal tissues, but also for the general well-being (Woelber et al. 2016).

## Concluding Remarks

It is worth mentioning that understanding periodontology as an immunological disorder has recently contributed to understanding the connection between periodontitis and systemic diseases, such as diabetes, cardiovascular disorders, preterm low birth weight, Alzheimer disease and others. In several of the mentioned conditions, periodontitis is considered as an established risk factor, there is a possibility of discovering new connections in the near future. Those associations are strictly related to immunological reaction, like in diabetes mellitus, where chronic oxidative stress cumulates and is believed to stand behind the aggravation of one disease by another one (Polak and Shapira 2018). In other conditions, like cardiac events or birth complications, the nature of the connection is not yet proven, but chronic inflammation and circulating immunomediators are commonly regarded as bridging factors (Fischer et al. 2021). The gastrointestinal pathway serves as a relatively easy transit route and may link periodontitis with a spectrum of bowel and liver diseases (Rinčić et al. 2022). The last 25 years have brought the paradigm shift from periodontitis regarded as an infection of the pocket to a hyper-reactive response of the immune system to such infection. Further advances in both periodontology and immunology will surely enhance possibilities in the field of prophylaxis, treatment and maintenance of the patients. Modulation of inflammatory response has an even more significant aim. Recent publications of American authors point at the patient’s affinity to develop periodontal disease as the symptom of fast path to chronic diseases, in opposition to smooth aging curve (Kornman and Giannobile 2017). Fast path patients tend to overload their immune system early with unhealthy habits, such as fatty and sugar-rich diet leading to obesity or smoking, which soon lead to development of diseases connected with disturbance of the immune system. The goal of each patient and physician should be to keep the patients on smooth aging curve, so that—according to the studies—they can reach old age in good physical and mental condition (Kornman and Giannobile 2017).
